# Vehicle Stability Analysis under Extreme Operating Conditions Based on LQR Control

**DOI:** 10.3390/s22249791

**Published:** 2022-12-13

**Authors:** Liping Wu, Ran Zhou, Junshan Bao, Guang Yang, Feng Sun, Fangchao Xu, Junjie Jin, Qi Zhang, Weikang Jiang, Xiaoyou Zhang

**Affiliations:** 1 School of Mechanical Engineering, Shenyang University of Technology, Shenyang 110870, China; 2 SIASUN Robot & Automation Co., Ltd., Shenyang 110169, China; 3 School of Mechanical Engineering, Shenyang Aerospace University, Shenyang 110136, China; 4 Department of Mechanical Engineering, Nippon Institute of Technology, Saitama 345-8501, Japan

**Keywords:** LQR controller, extreme operating conditions, vehicle stability, CarSim

## Abstract

Under extreme working conditions such as high-speed driving on roads with a large road surface unevenness coefficient, turning on a road with a low road surface adhesion coefficient, and emergency acceleration and braking, a vehicle’s stability deteriorates sharply and reduces ride comfort. There is extensive existing research on vehicle active suspension control, trajectory tracking, and control methods. However, most of these studies focus on conventional operating conditions, while vehicle stability analysis under extreme operating conditions is much less studied. In order to improve the stability of the whole vehicle under extreme operating conditions, this paper investigates the stability of a vehicle under extreme operating conditions based on linear quadratic regulator (LQR) control. First, a seven degrees of freedom (7-DOF) dynamics model of the whole vehicle is established based on the use of electromagnetic active suspension, and then an LQR controller of the electromagnetic active suspension is designed. A joint simulation platform incorporating MATLAB and CarSim was built, and the CarSim model is verified by real vehicle tests. Finally, the stability of the vehicle under four different ultimate operating conditions was analyzed. The simulation results show that the root mean square (RMS) values of body droop acceleration and pitch angle acceleration are improved by 57.48% and 28.81%, respectively, under high-speed driving conditions on Class C roads. Under the double-shift condition with a low adhesion coefficient, the RMS values of body droop acceleration, pitch acceleration, and roll angle acceleration are improved by 58.25%, 55.41%, and 31.39%, respectively. These results indicate that electromagnetic active suspension can significantly improve vehicle stability and reduce driving risk under extreme working conditions when combined with an LQR controller.

## 1. Introduction

The automobile has become a common mode of transportation in peoples’ lives. With the improvement in living standards, people put higher requirements on vehicle performance, and the suspension system is an important part that affects this performance. The suspension connects the wheels to the body and reduces the vibration transmitted to the vehicle by the road. However, the damping and stiffness of passive suspensions are not adjustable, can only achieve optimal performance under specific operating conditions, and cannot adapt to varying operating conditions [1]. Conversely, active suspension has adjustable stiffness and damping, which can effectively improve vehicle stability [2,3]. Electromagnetic suspension has fast response characteristics, good control characteristics, and easy decoupling control, and, as such, has become a research hotspot in the field of active suspension [4,5,6]. Research on electromagnetic active suspension mainly focuses on the feed characteristics of the actuator [7,8,9], control method research [10,11,12], and suspension performance [13,14].

Van der Sande, T. P. J. et al. [15] designed a new high-bandwidth control method for an electromagnetic active suspension system and simulated it in a 1:4-scale car model to improve comfort and handling. Li Z. et al. [16] proposed a multi-objective optimal control method for active suspension systems. An integrated electromechanical coupling model between motor electromagnetic excitation and transient vehicle dynamics is considered, and the Pareto solution set of optimal parameters is calculated using a particle swarm algorithm. The simulation analysis illustrates that vehicle ride comfort and road holding can be effectively improved. Ataei, M. [17] et al. investigated a hybrid electromagnetic suspension system. They evaluated the ride comfort, road holding, and regenerative power and performed a multi-objective optimization using a genetic algorithm. The simulation analysis results showed improvements in ride comfort and road holding and a significant increase in regenerative power. Kou F. [18] et al. proposed a hybrid actuator composed of a linear motor and a solenoid valve to study fault-tolerant control during the fault condition. The experimental results show that the root mean square (RMS) of the spring-mass acceleration can be effectively reduced in the fault-tolerant control state, which improves the dynamic performance of the suspension. Sun X. [19] et al. proposed a model predictive thrust control (MPTFC) method for linear motors with a switching frequency term in the evaluation function, which is investigated for an automotive active suspension system. Simulation results show that a linear motor suspension with MPTFC is able to generate the required force according to the body vibration. Wei W. [20,21] et al. proposed an electromagnetic actuator that simultaneously achieves vehicle vibration suppression and power recovery. Long G. [22] et al. proposed a motor-driven actuator and experimentally obtained a good energy-feeding effect. Ding R. [23] et al. proposed a hybrid electromagnetic suspension and studied its active control and energy feeding. Asadi E. [24] et al. proposed a hybrid electromagnetic damper and conducted structural optimization and energy-feeding experiments. Xie L. [25] et al. proposed an electromagnetic energy-feeding damper and investigated its energy-feeding characteristics. An energy-fed electromagnetic actuator was designed by R. Zhou [26,27] et al. The sensitivity of each physical and electrical parameter of the actuator to the power recovery was analyzed, and the power recovery efficiency of this was investigated. The self-powering technology of the energy-fed electromagnetic actuator was also studied. Yao M. [28] et al. designed a nonlinear electromagnetic energy harvester (EMEH) for automotive suspensions. The effect of structural parameters on the output characteristics of the nonlinear electromagnetic energy harvester was investigated. The results show that the higher the road class and the higher the vehicle speed, the better the output characteristics of the nonlinear electromagnetic energy harvester. Young I. [29] et al. designed an anti-jerk optimal predictive control method for active and semi-active suspension systems. This approach can reduce body jerk and improve ride comfort. The above research has greatly contributed to improvements in the actuator structure, control strategy, optimization of control parameters, and energy feeding of electromagnetic active suspensions, leading to the rapid development of the electromagnetic active suspension field. The research has also contributed to the application to the vehicle of electromagnetic actuators. However, most of these studies were based on vehicles working under conventional operating conditions.

Li Z. [30] et al. proposed a real-time controller for an electric vehicle with a four-wheel independent motor drive. It was shown that the controller could effectively improve the vehicle’s overall stability under extreme conditions and has good robustness when the vehicle mass or road adhesion coefficient is uncertain. Hang P. [31] et al. designed an active rear steering (ARS) control system. The active safety performance of the vehicle under extreme conditions was investigated. The results showed that the ARS system facilitated the active safety performance of human drivers. Liu G. [32] et al. proposed a control method for a vehicle lateral stability control system. The complex friction condition operating conditions were simulated and analyzed by joint simulation in MATLAB and CarSim. The results showed that the proposed control algorithm could improve the vehicle’s stability effectively. Sun X. [33] et al. proposed a new adaptive non-singular fast terminal sliding mode control scheme. The analysis of a vehicle in wet road conditions was carried out by TruckSim–Simulink co-simulation. The results show that the lateral stability of the vehicle is significantly improved. The above studies mainly focus on the vehicle’s dynamic performance and control strategies at extreme operating conditions. At the same time, fewer studies have investigated the electromagnetic active suspension of a vehicle at extreme operating conditions. Ji Y. [34] et al. conducted a stability study of a hybrid electromagnetic suspension with an active lateral stabilizer bar. They showed through simulation that the suspension of this structure can improve the vehicle’s anti-roll performance.

In this paper, electromagnetic active suspension is studied. The performance of electromagnetic active suspension is investigated under extreme conditions, such as high-speed driving conditions on Class C roads, double-shift line conditions with low coefficient of adhesion, and emergency acceleration and braking conditions. A linear quadratic regulator (LQR) controller was designed, and the overall vehicle stability was analyzed using joint simulation in MATLAB and CarSim. Vehicle stability of the electromagnetic active suspension under extreme conditions is discussed, and the root mean square values of the main evaluation indexes of the electromagnetic active suspension under extreme conditions of operation are described. The novelty of this paper is that electromagnetic active suspension is studied for vehicle stability under extreme working conditions. A reference is provided for the performance of vehicles equipped with electromagnetic active suspension under extreme conditions.

This paper consists of six sections. In Section 2, the mathematical model of the vehicle is introduced. The LQR controller is given in Section 3. In Section 4, a joint MATLAB and CarSim simulation platform is built and verified by real vehicle tests. Four typical extreme operating conditions are also designed. Simulation analysis is performed in Section 5. Conclusions are given in Section 6.

## 2. Mathematical Modeling of Vehicles

**Figure 1:**
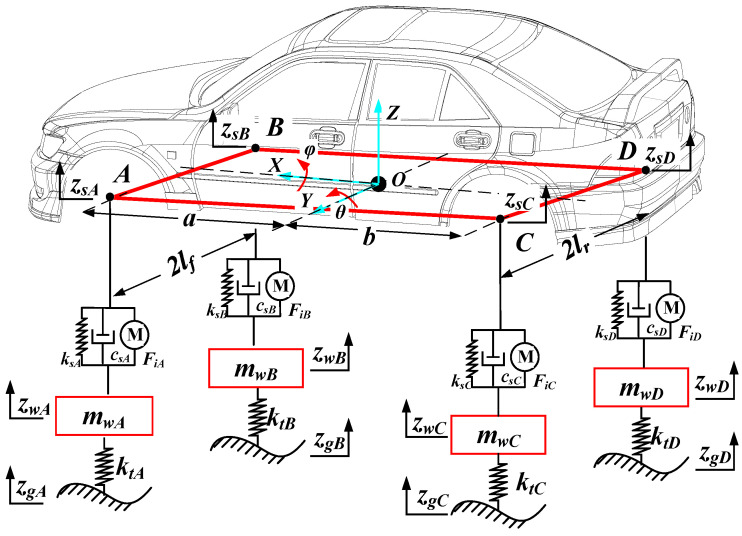
Schematic of the 7-DOF full-car suspension system.

The Cartesian coordinate system O-XYZ is established with the center of mass of the body as the origin O, the forward direction of the vehicle as the positive X-axis direction, and the direction of the body away from the ground as the positive Z-axis direction. A is the simplified connection point between the body and the left front suspension, B is the simplified connection point between the body and the right front suspension, C is the simplified connection point between the body and the left rear suspension, and D is the simplified connection point between the body and the right rear suspension. The wheels are simplified as linear springs with stiffness *k_ti_*, the suspension is simplified as springs with stiffness *k_si_* and dampers with damping *c_si_*, the wheels are simplified as unsprung masses with mass *m_wi_*, and finally, the vehicle is simplified as a seven degrees of freedom (7-DOF) model, as shown in Figure 1, containing the center-of-mass motion, pitch motion, lateral tilt motion, and vertical motion of the four suspensions of the vehicle.

Descriptions and units of each parameter in Figure 1 are shown in Table 1.

The vehicle’s kinematic equations are established according to Newton’s laws of motion. The equation of the body mass vertical motion along the Z-axis is:

(1) $$\begin{array}{c} m\ddot{z}=\sum_{j=A}^{D}-c_{sj}\left({\dot{z}}_{sj}-{\dot{z}}_{wj}\right)+\sum_{j=A}^{D}-k_{sj}\left(z_{sj}-z_{wj}\right)+\sum_{i=A}^{D}F_{ij}\\ j=A,\;B,\;C,\;D \end{array}$$

The equation of lateral tilt motion for rotation about the Y-axis is:

(2) $$I_{p}\ddot{\theta }=\sum^{\;}F_{f}\cdot a-\sum^{\;}F_{r}\cdot b\;\Sigma F_{f}=\overset{B}{\underset{j=A}{\sum }}-c_{sj}\left({\dot{z}}_{sj}-{\dot{z}}_{wj}\right)+\overset{B}{\underset{j=A}{\sum }}-k_{sj}\left(z_{sj}-z_{wj}\right)+F_{ij}\;\Sigma F_{r}=\overset{D}{\underset{j=C}{\sum }}-c_{sj}\left({\dot{z}}_{sj}-{\dot{z}}_{wj}\right)+\overset{D}{\underset{j=C}{\sum }}-k_{sj}\left(z_{sj}-z_{wj}\right)+F_{ij}\;j=A,\;B,\;C,\;D$$

where *I_p_* is the rotational inertia of the body to the Y axis, ∑*F_f_* is the combined forces of the front suspension, and ∑*F_r_* is the combined forces of the rear suspension.

The equation of lateral tilt motion for rotation about the X-axis is:

(3) $$I_{r}\ddot{\varphi }=\left(\sum^{F_{A}}-\sum^{F_{B}}\right)\cdot t_{f}+\left(\sum^{F_{C}}-\sum^{F_{D}}\right)\cdot t_{r}\;\Sigma F_{j}=-c_{sj}\left({\dot{z}}_{sj}-{\dot{z}}_{wj}\right)-k_{sj}\left(z_{sj}-z_{wj}\right)+F_{ij}\;j=A,\;B,\;C,\;D$$

where *I_r_* is the rotational inertia of the body to the X-axis, ∑*F_A_* is the combined forces of the left front suspension, ∑*F_B_* is the combined forces of the rear front suspension, ∑*F_C_* is the combined forces of the left rear suspension, and ∑*F_D_* is combined forces of the right rear suspension.

The equations of vertical motion of the four suspensions are:

(4) $$\begin{array}{c} m_{wj}{\ddot{z}}_{wj}=c_{sj}\left({\dot{z}}_{sj}-{\dot{z}}_{wj}\right)+k_{sj}\left(z_{sj}-z_{wj}\right)-k_{tj}\left(z_{wj}-z_{gj}\right)+F_{ij}\\ j=A,\;B,\;C,\;D \end{array}$$

## 3. Research of Control Strategy

**Figure 2:**
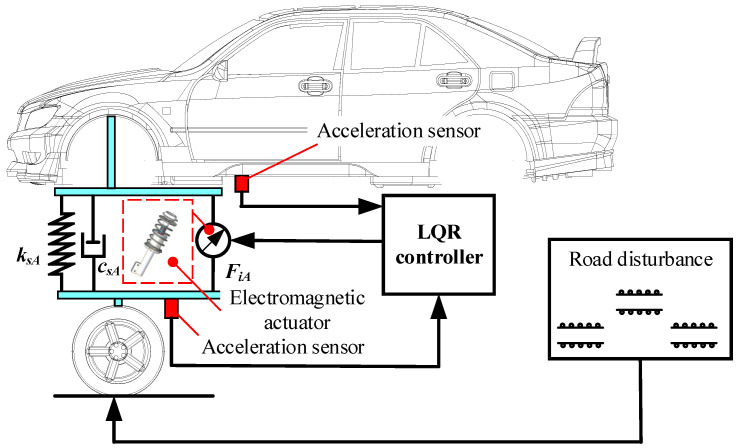
Schematic of the electromagnetic active suspension with LQR controller.

Vehicle stability is mainly reflected in ride comfort, driving smoothness, and handling stability, and the suspension system is an important element in vehicle stability. The suspension system in the body’s vertical acceleration, suspension dynamic deflection, and dynamic tire load can reflect, to a certain extent, the ride comfort, smoothness, handling stability, etc. Therefore, the controller design aims to reduce the body droop acceleration and dynamic tire load and keep the suspension dynamic deflection in a reasonable range. In extreme operating conditions, the body vibration and attitude of the whole vehicle must also be considered to ensure its safety. This paper uses the LQR control strategy to analyze overall vehicle stability under extreme operating conditions. A schematic of the electromagnetic active suspension with the LQR controller is shown in Figure 2.

### 3.1. State Space Equations for Electromagnetic Active Suspension

According to equations (1)–(4), appropriate state quantities and input and output quantities are selected to establish the state space equations. Table 2 lists the state variables and input and output quantities of the whole vehicle.

According to the state quantity, input quantity, and output quantity selected in Table 1, the state equation can be obtained as follows:

(5) $$\{\begin{array}{c} \dot{X}=AX+BU\\ Y=CX+DU \end{array}$$

where *X* is the state variable matrix, *U* is the input variable matrix, *A* is the coefficient matrix of the system, *B* is the input coefficient matrix, *C* is the output coefficient matrix, and *D* is the transfer matrix.

### 3.2. Design of LQR Controller for Electromagnetic Active Suspension

The aim of LQR control is to achieve effective control of each control objective at a small expense. Control of the vehicle suspension system is mainly aimed at improving vehicle ride comfort, handling stability, and smoothness. Thus, the performance indicators reflected in the suspension system are mainly the body’s vertical acceleration, the suspension’s dynamic travel, and the tires’ dynamic displacement. The pitch and roll of the vehicle also need to be considered in the whole vehicle analysis. According to the above analysis, the evaluation model of electromagnetic active suspension LQR is established with body droop acceleration, pitch angle acceleration, side camber acceleration, four suspension dynamic travels, four dynamic tire displacements, and four suspension active forces. As in Equation (6):

(6) $$J=\underset{T\to \infty }{\lim}\frac{1}{T}\int_{0}^{T}(\begin{array}{c} q_{1}\ddot{z}+q_{2}\ddot{\theta }+q_{3}\ddot{\varphi }+q_{4}{\left(z_{sA}-z_{wA}\right)}^{2}+q_{5}{\left(z_{sB}-z_{wB}\right)}^{2}\\ +q_{6}{\left(z_{sC}-z_{wC}\right)}^{2}+q_{7}{\left(z_{sD}-z_{wD}\right)}^{2}+q_{8}{\left(z_{wA}-z_{gA}\right)}^{2}\\ +q_{9}{\left(z_{wB}-z_{gB}\right)}^{2}+q_{10}{\left(z_{wC}-z_{gC}\right)}^{2}+q_{11}{\left(z_{wD}-z_{gD}\right)}^{2}\\ +r_{1}F_{A}^{2}+r_{2}F_{B}^{2}+r_{3}F_{C}^{2}+r_{4}F_{D}^{2} \end{array})dt$$

where *q_j_* (*j* = 1,2…,11) is the weighting factor, $\ddot{z}$ is the vertical body acceleration, $\ddot{\theta }$ is the pitch acceleration, $\ddot{\varphi }$ is the side camber acceleration, *z_sj_*- *z_wj_* (*j* = A, B, C, D) is the *j* suspension dynamic travel, *z_wj_*- *z_gj_* (*j* = A, B, C, D) is the *j* dynamic tire deformation, and *F_j_* (*j* = A, B, C, D) is the *j* suspension main force.

The weight matrix is taken as:

(7) $$\begin{array}{c} q=diag(q_{1},q_{2}\cdots q_{11})\\ r=diag(r_{1},r_{2},r_{3},r_{4}) \end{array}$$

The performance panacea of Eq. (6) can be written as:

(8) $$J=\underset{T\to \infty }{\lim}\frac{1}{T}\int_{0}^{T}\left(Y^{T}qY+F^{T}rF\right)dt\;=\underset{T\to \infty }{\lim}\frac{1}{T}\int_{0}^{T}\left[{\left(CX+DF\right)}^{T}q\left(CX+DF\right)+F^{T}rF\right]dt\;=\underset{T\to \infty }{\lim}\frac{1}{T}\int_{0}^{T}\left[X^{T}C^{T}qCX+2X^{T}C^{T}qDF+F\left(D^{T}qD+r\right)F\right]dt$$

Then the weighting matrix of the state variables can be expressed as:

(9) $$Q=C^{T}qC$$

The weighting matrix of the control variables is:

(10) $$R=D^{T}qD+r$$

The Riccati equation leads to *P*:

(11) $$A^{T}P+PA+Q-PBR^{-1}B^{T}P=0$$

A further solution of P obtained from equation (11) yields the feedback matrix *K* as:

(12) $$K=-R^{-1}BP$$

*U* can be expressed as:

(13) U=−KX

## 4. Simulation Platform Construction

### 4.1. Joint Simulation of CarSim and Simulink

A joint simulation with MATLAB/Simulink and the vehicle simulation software CarSim is used to investigate the effect of the LQR control strategy. The input variables are shown in Table 3. And the output variables are shown in Table 4.

**Figure 3:**
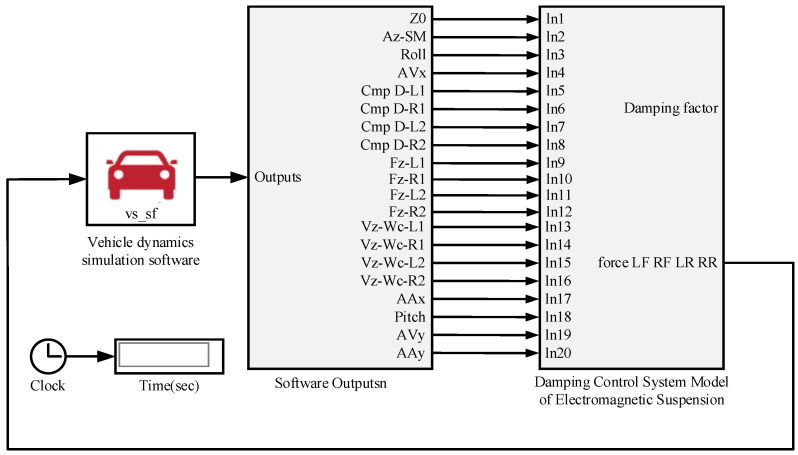
Block diagram of CarSim and Simulink joint simulation.

The joint simulation block diagram is shown in Figure 3. The vehicle dynamics simulation software CarSim provides the vehicle state parameters and Simulink provides the parameters of the electromagnetic active suspension actuator and replaces the original actuator parameters in the vehicle dynamics in CarSim to realize the simulation analysis of the LQR control of the electromagnetic active suspension system.

### 4.2. Design of the Simulation Conditions

Our aim is to investigate the vehicle’s stability under extreme working conditions. The extreme working condition model is established in CarSim vehicle dynamics simulation software.

**Figure 4:**
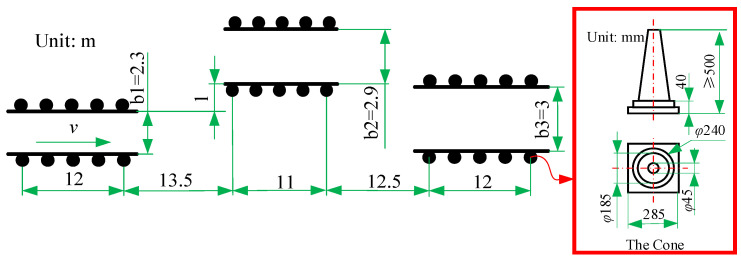
Schematic diagram of the location of the double shift line test pile.

1. A double shift line test is generally used to evaluate vehicle handling stability, as outlined in ISO3888-3-2011 (E). The double shift line test condition arrangement is carried out, as shown in Figure 4.

Where the width of the road is calculated by Equation (14).

(14) $$\begin{array}{l} b_{1}=1.1\times b_{vehicle}+0.25\\ b_{2}=b_{vehicle}+1\\ b_{3}=1.3\times b_{vehicle}+0.25,b_{3}\geq 3 \end{array}$$

where *b_vehicle_* is the vehicle width, taken to be 1.855 m, so the calculated *b*_1_ is 2.2905 m, taken to be 2.3 m, *b*_2_ is 2.855 m, taken to be 2.9 m, *b*_3_ is 2.6615 m; however, because *b*_3_ cannot be less than 3 m, *b*_3_ is taken to be 3 m.

The direction of travel is shown in Figure 4, and the driving speed is taken as 60 km/h with a road adhesion coefficient of 0.5.

2. According to the introduction in ISO 2631-1: 1997, automobile smoothness test method, the speed of the car in the good road surface test can reach the maximum design speed, while the speed of the general road surface test generally does not exceed 70 km/h. Our aim is to explore the car’s smoothness when driving at high speed on a poorer road surface, and the Class C road surface is chosen with a speed of 120 Km/h. The Class C road surface is expressed in Equation (15).
  (15) $${\dot{z}}_{g}\left(t\right)=-2\pi f_{0}z_{g}\left(t\right)+2\pi \sqrt{G_{0}u}\cdot w\left(t\right)$$
  where *x_g_* (*t*) is the white noise road random excitation, *w* (*t*) is the white noise with the mean value of 0, *G_0_* is the road unevenness coefficient (256 × 10^−6^ m^3^ for a C-class road), and *f*_0_ is the cut-off frequency (0.06 Hz for a general random road).

**Figure 5:**
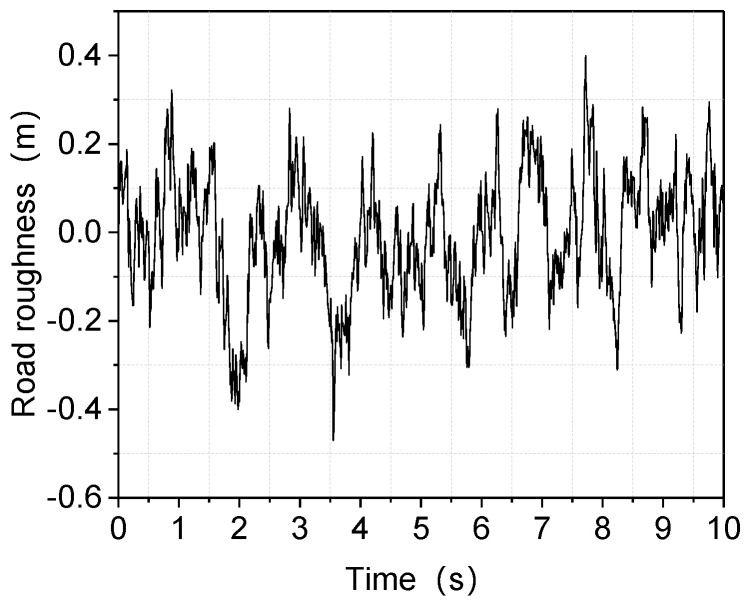
Obtained random road excitation for a Class C road.

From equation (15), the obtained random road excitation is shown in Figure 5.

3. Emergency acceleration and braking will greatly affect vehicle stability. According to the requirements of GB21670-2008, the design of emergency acceleration and braking conditions, the specific needs are for the vehicle to accelerate from a standstill to 100 km/h within 5 s. Then the speed is stabilized at 100 km/h before the emergency braking.

### 4.3. Verification of the Whole Vehicle Model

The accuracy of the vehicle model in the vehicle dynamics software—CarSim—is important for the subsequent analysis. To verify the accuracy of the vehicle model in CarSim, a real vehicle road experiment is conducted to validate the vehicle parameters in CarSim. The vehicle parameters refer to a Toyota RAV4, as shown in Table 5.

**Figure 6:**
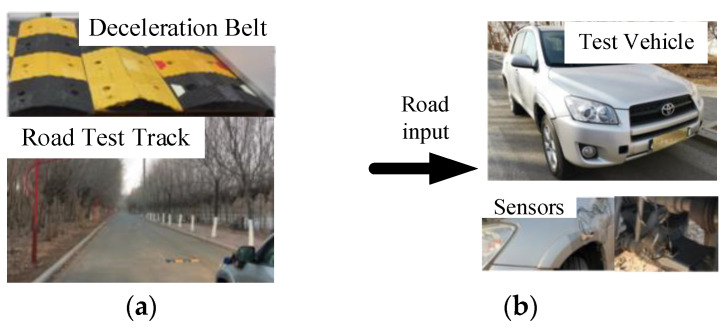

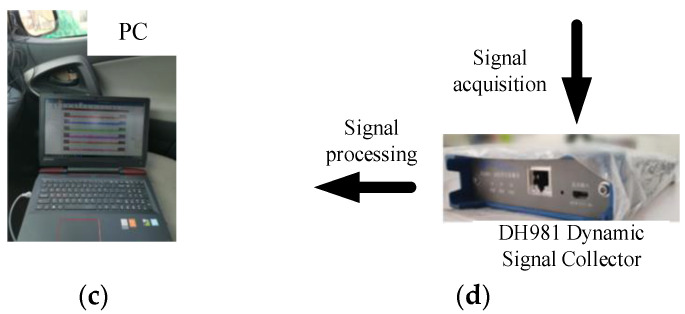
Road test of the vehicle. (**a**) vehicle test ground; (**b**) experimental vehicle; (**c**) computer; (**d**) dynamic signal collector.

The vehicle road real-world experiment is shown in Figure 6. Piezoelectric acceleration sensors (Donghua 1A302E-IEPE, Taizhou City, Jiangsu Province, China) are fixed on both sides of the body’s lower swing arm to collect vibration signals. A distributed dynamic signal test and analysis system (DH5981, Taizhou City, Jiangsu Province, China) was used to detect and analyze the vibration signals. The accuracy of the vehicle model when the vehicle passed over different speed bumps was tested.

**Figure 7:**
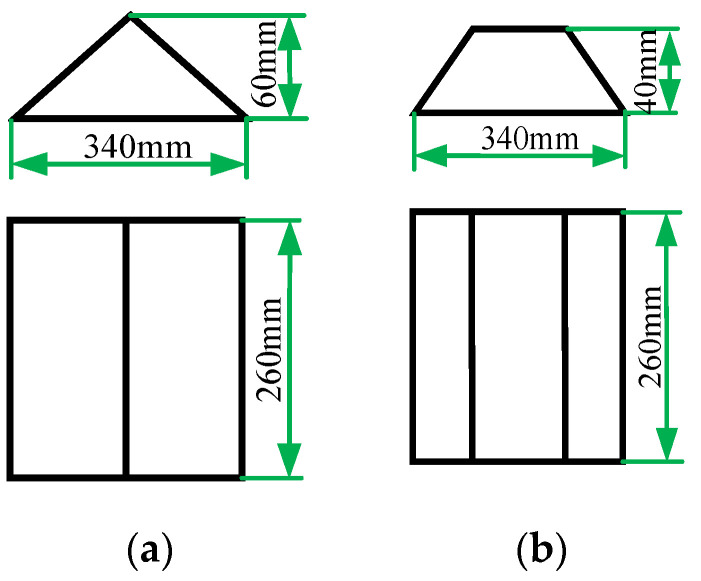
Diagram of speed reduction belt. (**a**) triangular speed reduction belt; (**b**) trapezoidal speed reduction belt.

A 200 m section of straight B-grade road was selected as the experimental road, and triangular speed bumps and trapezoidal speed bumps were selected for separate testing. The schematic diagram of the speed bumps is shown in Figure 7.

**Figure 8:**
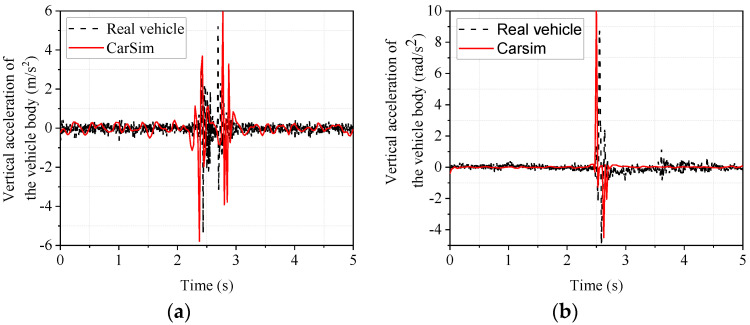
Response of the body when passing over a triangular speed bump. (**a**) vertical acceleration of the body; (**b**) lateral camber acceleration of the body.

The experimental car passed over the speed bump at a constant speed of 20 km/h, and the same working condition, speed, and speed bump were set in CarSim. Figure 8 shows the acceleration responses of vertical body vibration and body roll angle when the vehicle passes over the triangular speed bump.

From Figure 8, it can be seen that the trends of the real vehicle and the CarSim model are the same in the body droop acceleration and body side camber acceleration. The front wheels and rear wheels passing over the speed bump in turn cause the two sudden signal changes in Figure 8a. In order to analyze the accuracy of the vehicle dynamics software modeling more intuitively, the data in Figure 8 are taken as the maximum and minimum values for comparison and analysis. The results are shown in Table 6.

Since the peak value better reflects the vehicle’s state due to the bumpy road [35], the maximum and minimum values are analyzed. As seen from Table 6, the maximum values of the body’s vertical and lateral camber acceleration are 12.61% and 15.45%, respectively, and the minimum values are 7.31% and 7.74%, respectively, which are not significant. This indicates that the CarSim model can accurately reflect the real car.

**Figure 9:**
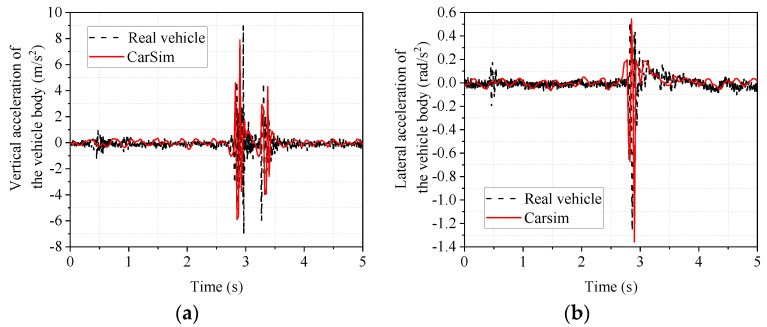
Trapezoidal speed bump body response. (**a**) vertical acceleration of the body; (**b**) lateral camber acceleration of the body.

The acceleration responses of vertical body vibration and body roll angle when the vehicle passes over the trapezoidal speed bump are shown in Figure 9.

From Figure 9, it can be seen that the trends of the real vehicle and the CarSim model are the same in the body droop acceleration and side camber acceleration, and the front and rear wheels passing over the speed bump, in turn, cause the two sudden signal changes in Figure 9a. In order to analyze the accuracy of the vehicle dynamics software CarSim modeling more intuitively, the data in Figure 9 were analyzed, and the maximum and minimum values were taken for comparison. The results are shown in Table 7.

As seen from Table 7, the maximum values of the body’s vertical and lateral camber acceleration are 14.04% and 18.75%, respectively, and the minimum values are 3.41% and 6.81%, respectively, which are not significant. This again indicates that the CarSim model can accurately reflect the real car.

## 5. Analysis of Simulation Results

### 5.1. Simulation Analysis of High-speed Driving Conditions on Class C Roads

**Figure 10:**
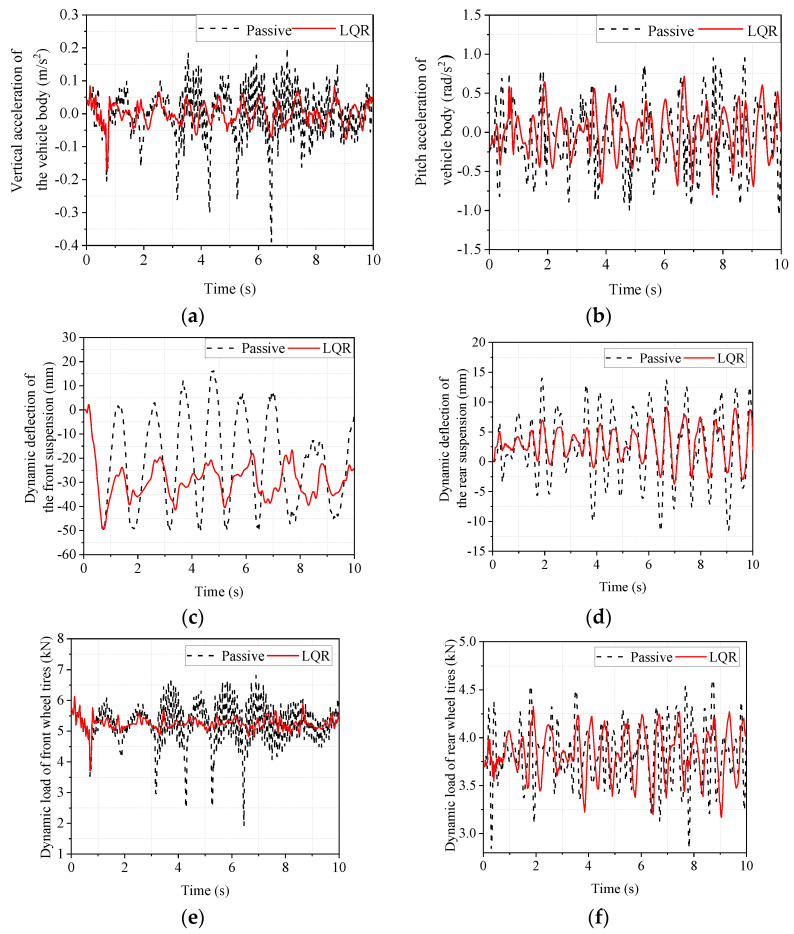
Simulated time domain response of high-speed conditions on Class C roads. (**a**) body vertical acceleration; (**b**) body pitch angle acceleration; (**c**) front suspension dynamic deflection; (**d**) rear suspension dynamic deflection; (**e**) front wheel tire dynamic load; (**f**) rear wheel tire dynamic load.

In order to investigate the smoothness of a vehicle driving at high speed on a general road surface, a simulation analysis of high-speed driving conditions on a Class C road surface was conducted. The vehicle’s driving speed is set to 120 km/h, and the simulation results are shown in Figure 10. From Figure 10, it can be seen that the LQR-controlled electromagnetic active suspension has improved body droop acceleration, body pitch acceleration, dynamic deflection of front and rear suspension, and dynamic load of the front and rear tires compared with the passive suspension. Since the vehicle is driven in a straight line in the CarSim simulation, the left and right sides are symmetrical, so the left and right suspensions are not distinguished, and only the response of the front and rear suspensions are analyzed. Similarly, since the vehicle is a symmetrical structure, the side camber angle is very small in the straight-line driving condition, so the image of the side camber angle is not presented in Figure 10.

In order to objectively analyze the improvement in the suspension, the maximum, minimum, and RMS values were calculated for the responses in Figure 10 and analyzed for comparison. The results are shown in Table 8.

The RMS values reflect the performance of the vehicle suspension on random road surfaces. As can be seen from Table 8, the LQR-controlled electromagnetic active suspension improved the acceleration of body droop by 57.48%, the acceleration of pitch angle by 28.81%, and the dynamic deflection of the rear suspension by 34.78% compared with the passive suspension. In contrast, the front tires’ dynamic load and the rear tires’ dynamic load did not change much, while the dynamic deflection of the front suspension deteriorated by 9.24%. Although the dynamic deflection of the front suspension deteriorated by 9.24%, the improvement in body droop acceleration and body pitch angle acceleration, which affect the smoothness more, were both larger, indicating that the LQR-controlled electromagnetic suspension can better improve the smoothness of the vehicle under high-speed driving conditions on general roads.

### 5.2. Simulation Analysis of Double-Shifted Line Working Condition with Low Adhesion Coefficient

**Figure 11:**
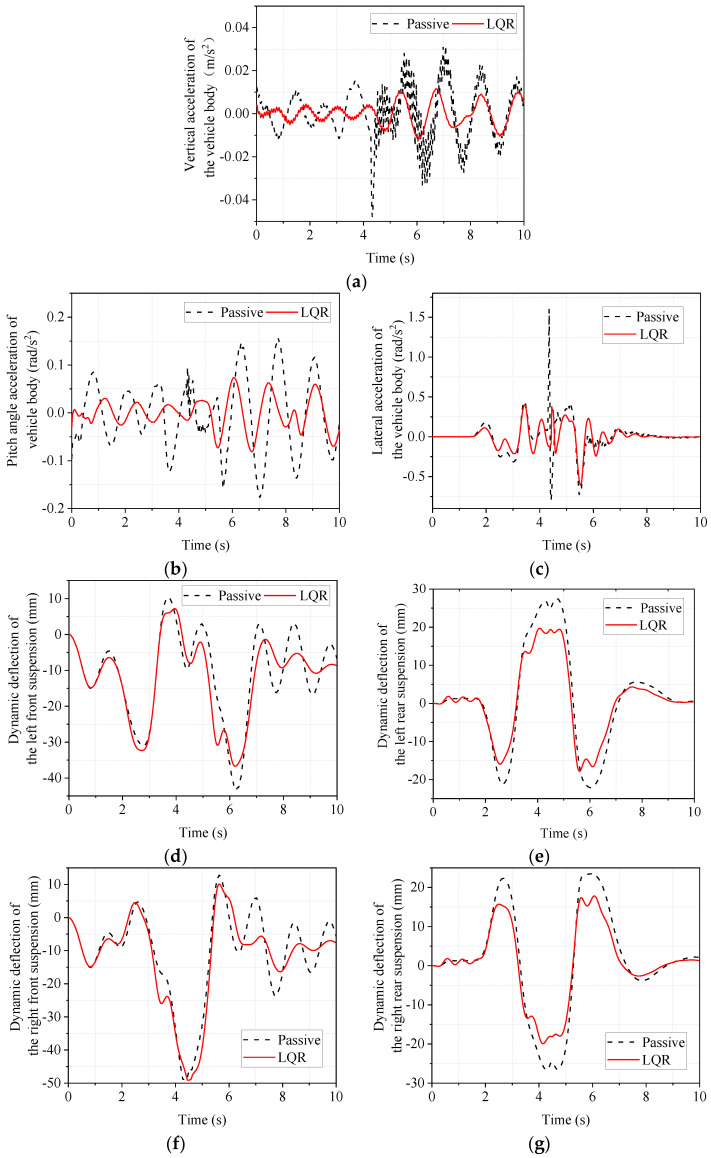

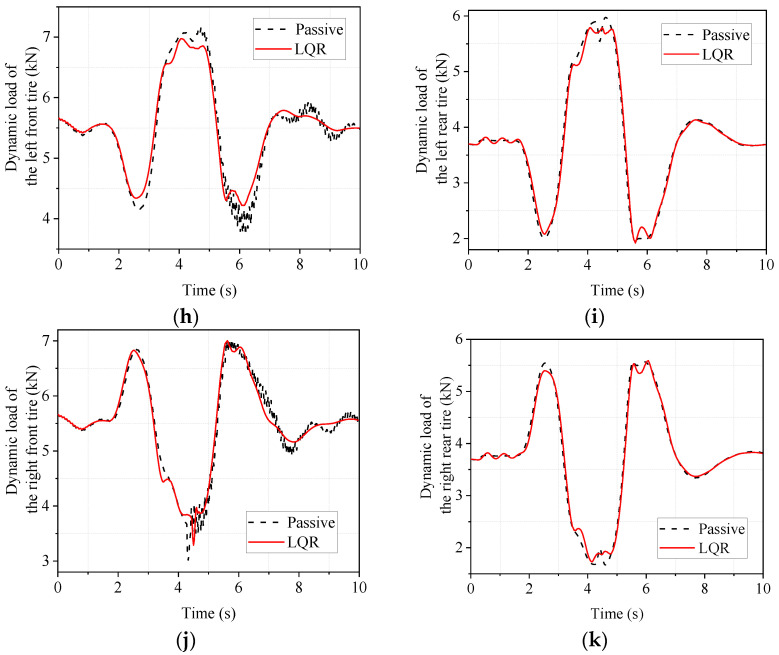
Simulated time domain response of low adhesion coefficient double shift line condition. (**a**) body vertical acceleration; (**b**) body pitch acceleration; (**c**) body lateral camber acceleration; (**d**) left front suspension dynamic deflection; (**e**) left rear suspension dynamic deflection; (**f**) right front suspension dynamic deflection; (**g**) right rear suspension dynamic deflection; (**h**) left front tire dynamic load; (**i**) left rear tire dynamic load; (**j**) right front tire dynamic load; (**k**) right rear tire dynamic load.

The double-shift emergency lane change operation is a test in which the vehicle is driven from one lane to another and back to the original lane. It is a dynamic evaluation that can reflect the vehicle’s stability by measurement of the side deflection angle of the vehicle center of mass, body droop acceleration, and other indicators. The road surface adhesion coefficient also affects the stability of the vehicle. In order to investigate the limit state of the vehicle, a double-shift working condition test with a low adhesion coefficient is therefore designed. The vehicle’s driving speed is set to 60 km/h, the road adhesion coefficient is set to 0.5, and the simulation results are shown in Figure 11. As shown in Figure 11, the LQR-controlled electromagnetic active suspension has improved body droop acceleration, body pitch acceleration, body roll acceleration, dynamic deflection of four suspensions, and dynamic load of four tires compared to the passive suspension.

The maximum, minimum, and RMS values of the responses in Figure 11 are calculated and compared to objectively analyze the degree of improvement. The results are shown in Table 9.

As seen from Table 9, the LQR-controlled electromagnetic active suspension has a greater improvement in body droop acceleration, body pitch angle acceleration, and body roll angle acceleration, both in terms of RMS and peak values, compared to the passive suspension. This indicates that the LQR-controlled electromagnetic active suspension can effectively improve the smoothness of the vehicle under the double-shift condition with a low adhesion coefficient. As for the more important tire dynamic load of vehicle road adhesion, the RMS values of dynamic tire load of the left front wheel, left rear wheel, right front wheel, and right rear wheel improved −0.15%, 0.37%, −0.02%, and 0.37%, respectively, with no significant difference. As for the absolute value of the peak, the left front wheel, left rear wheel, and right front wheel improved by 2.75%, 3.05%, and 0.49%, respectively, all slightly, while there was almost no change in the dynamic load of the right rear wheel. Therefore, the LQR-controlled electromagnetic active suspension does not deteriorate for road holding in double-shift conditions with a low adhesion coefficient. At the same time, it also effectively improves vehicle ride comfort.

### 5.3. Simulation Analysis of Emergency Acceleration and Braking Conditions

**Figure 12:**
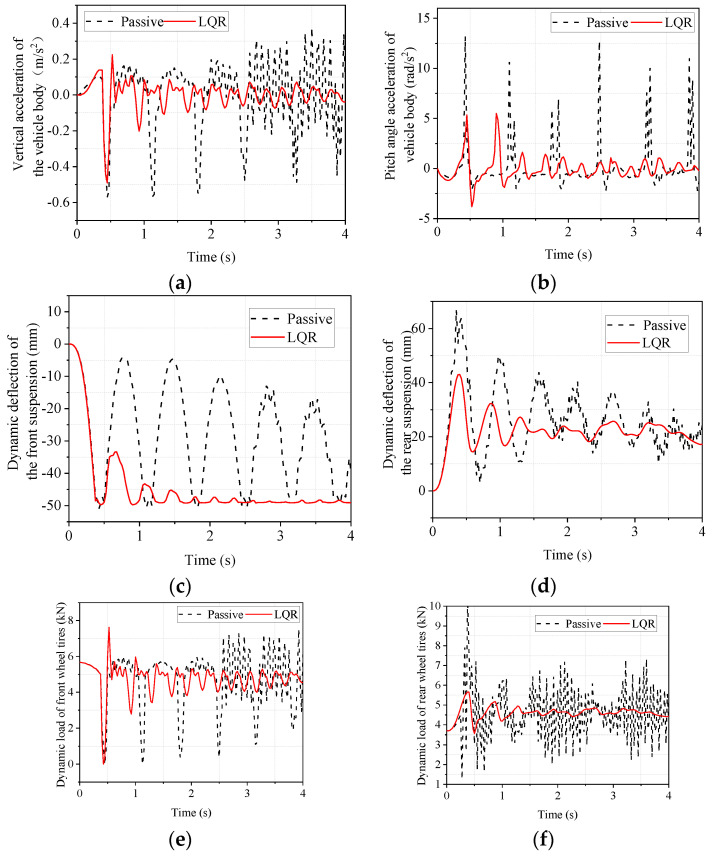
Simulated time domain response of emergency acceleration condition. (**a**) body vertical acceleration; (**b**) body pitch angle acceleration; (**c**) front suspension dynamic deflection; (**d**) rear suspension dynamic deflection; (**e**) front wheel tire dynamic load; (**f**) rear wheel tire dynamic load.

Vehicle acceleration and braking are important indicators of the performance of a vehicle, and a vehicle in the acceleration and braking phase is also the most likely to cause vibration and bumps. So, to explore the emergency acceleration and braking conditions of the vehicle ride comfort and road holding are very important. In CarSim, we set the vehicle to accelerate from 0 to 100 km/h in 5 s. After the vehicle speed stabilizes at 100 km/h, braking starts. In order not to affect the analysis of the acceleration phase during the braking phase, 0–4 s is taken as the acceleration phase for analysis, and 6–10 s is taken as the deceleration phase for analysis. The time domain response of the acceleration phase is shown in Figure 12. It can be seen from Figure 12 that the LQR-controlled electromagnetic active suspension improves body droop acceleration, body pitch angle acceleration, dynamic deflection of the front and rear suspensions, and dynamic loads on the front and rear tires compared to the passive suspension. Since the vehicle is driven in a straight line in the CarSim simulation, the left and right sides are symmetrical, so the left and right suspensions are not distinguished, and only the response of the front and rear suspensions are analyzed. Similarly, since the vehicle is a symmetrical structure, the side camber angle is very small in the straight-line driving condition, so the image of the side camber angle is not presented in Figure 12.

The maximum, minimum, and RMS values of the responses in Figure 12 are calculated and compared to objectively analyze the degree of improvement. The results are shown in Table 10.

As can be seen in Table 10, there is some deterioration in the RMS value of the front suspension dynamic deflection and little change in the dynamic deflection of the rear suspension and dynamic load of the front and rear tires. The RMS values of body droop and pitch angle acceleration have improved considerably. This indicates that, although the index of a particular suspension deteriorates, the LQR-controlled electromagnetic active suspension can effectively improve ride comfort during acceleration from the perspective of the whole vehicle.

**Figure 13:**
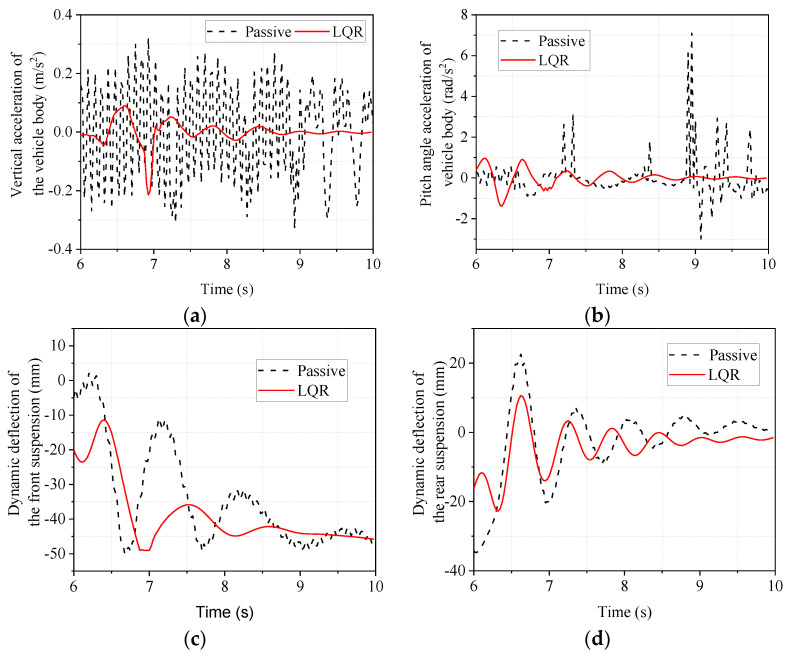

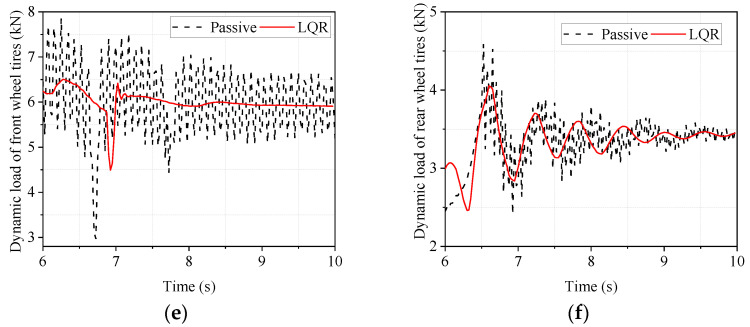
Simulated time domain response of emergency braking condition. (**a**) body vertical acceleration; (**b**) body pitch angle acceleration; (**c**) front suspension dynamic deflection; (**d**) rear suspension dynamic deflection; (**e**) front wheel tire dynamic load; (**f**) rear wheel tire dynamic load.

The time-domain response of the deceleration phase is shown in Figure 13. From Figure 13 it can be seen that the LQR-controlled electromagnetic active suspension has improved body droop acceleration, body pitch angle acceleration, front and rear suspension dynamic deflection, and front and rear tire dynamic loads compared to the passive suspension.

The maximum, minimum, and RMS values of the responses in Figure 13 are calculated and compared to objectively analyze the degree of improvement. The results are shown in Table 11.

As is shown in Table 11, the RMS values of the dynamic tire loads of the front and rear wheels are almost unchanged. The RMS value of rear suspension dynamic deflection improved by 35.91%, but the RMS value of front suspension dynamic deflection deteriorated by 7.48%. Concerning the acceleration of the body in the vertical direction and the acceleration of the pitch angle, it can be seen that the LQR-controlled electromagnetic active suspension improves these by 76.39% and 64.44%, respectively, compared to the passive suspension. Even though the improvement in other indicators is not obvious, the improvement in body droop acceleration and pitch angle acceleration, which reflect vehicle ride comfort, is more obvious, indicating that the LQR-controlled electromagnetic active suspension can significantly improve the ride comfort of the vehicle.

## 6. Conclusions

In this paper, firstly, a 7-DOF vehicle dynamics model is established, and an LQR controller is designed. Secondly, the general road high-speed driving condition, low adhesion coefficient double shift line condition, emergency acceleration condition, and emergency braking condition are designed. Then, the simulation of LQR control with electromagnetic suspension and passive suspension is carried out by the joint simulation using CarSim vehicle dynamics software and Matlab/Simulink. The indexes are compared and analyzed. The following conclusions can be drawn.

1. In the high-speed driving condition on a Class C road, the LQR-controlled electromagnetic active suspension significantly improved the vehicle body droop acceleration and body pitch angle acceleration by 57.48% and 28.81%, respectively. While the front suspension dynamic deflection deteriorated by 9.24% and the rear suspension dynamic deflection improved by 34.78%, the front, and rear tire dynamic load did not change much. This shows that the LQR-controlled electromagnetic active suspension can effectively improve the ride comfort of the whole vehicle.
2. In the double-shift line condition with a low adhesion coefficient, the LQR-controlled electromagnetic active suspension improved the RMS values of the vehicle’s body droop acceleration, body pitch angle acceleration, and body roll angle acceleration by 58.25%, 55.41%, and 31.39%, respectively; and the peak values by 75.63%, 54.11%, and 74.88%, respectively. There is almost no significant change in its dynamic tire load. The LQR-controlled electromagnetic active suspension effectively improves vehicle ride comfort without deteriorating road holding.
3. In the emergency acceleration condition, the RMS value of the front suspension dynamic deflection of the vehicle has some deterioration. Still, its peak value has improved, indicating that the LQR-controlled electromagnetic active suspension can effectively avoid collision with the limiting block. In addition, the RMS values of body droop acceleration and pitch angle acceleration improved by 58.54% and 54.13%, respectively. This shows that LQR-controlled electromagnetic active suspension can effectively improve ride comfort.
4. In the emergency braking condition, the RMS value of the front suspension dynamic deflection of the vehicle deteriorated by 7.48%, but its peak value improved by 15.32%, which can effectively prevent the suspension from colliding with the limiting block. The body droop and pitch angle acceleration improved by 76.39% and 64.44%, respectively. Thus, the LQR-controlled electromagnetic active suspension can improve vehicle ride comfort.

This paper presents a vehicle stability study of an LQR-controlled electromagnetic active suspension under extreme operating conditions. The results demonstrate that this control strategy can ensure vehicle stability during extreme operating conditions. This paper’s research results can provide a reference for the lateral stability of vehicles equipped with electromagnetic active suspension systems. In the future, we will design an electromagnetic active suspension system based on the results of this paper. We will study the coupling and control between lateral and longitudinal stability of the whole vehicle electromagnetic active suspension, measure the displacement and acceleration of the suspension using acceleration and laser displacement sensors, and carry out research related to the whole vehicle test verification of the electromagnetic active suspension and in-depth discussion and study of control strategy managerial points.

## Figures and Tables

**Table 1:** 7-DOF vehicle parameters.

Description	Symbols	Unit	Description	Symbols	Unit
Left front wheel tire stiffness	*k_tA_*	N/m	Left front wheel road excitation	*z_gA_*	m
Right front wheel tire stiffness	*k_tB_*	N/m	Right front wheel road excitation	*z_gB_*	m
Left rear wheel tire stiffness	*k_tC_*	N/m	Left rear wheel road excitation	*z_gC_*	m
Right rear wheel tire stiffness	*k_tD_*	N/m	Right rear wheel road excitation	*z_gD_*	m
Left front suspension stiffness	*k_sA_*	N/m	Left front un-sprung mass displacement	*z_wA_*	m
Right front suspension stiffness	*k_sB_*	N/m	Right front un-sprung mass displacement	*z_wB_*	m
Left rear suspension stiffness	*k_sC_*	N/m	Left rear un-sprung mass displacement	*z_wC_*	m
Right rear suspension stiffness	*k_sD_*	N/m	Right rear un-sprung mass displacement	*z_wD_*	m
Left front suspension damping	*c_sA_*	Ns/m	Left front sprung mass displacement	*z_sA_*	m
Right front suspension damping	*c_sB_*	Ns/m	Right front sprung mass displacement	*z_sB_*	m
Left rear suspension damping	*c_sC_*	Ns/m	Left rear sprung mass displacement	*z_sC_*	m
Right rear suspension damping	*c_sD_*	Ns/m	Right rear sprung mass displacement	*z_sD_*	m
Left front un-sprung mass	*m_wA_*	kg	Left front suspension main force	*F_iA_*	N
Right front un-sprung mass	*m_wB_*	kg	Right front suspension main force	*F_iB_*	N
Left rear un-sprung mass	*m_wC_*	kg	Left rear suspension main force	*F_iC_*	N
Right rear un-sprung mass	*m_wD_*	kg	Left rear suspension main force	*F_iD_*	N
Distance from the front wheel to the center of mass	*a*	mm	Vertical displacement of the vehicle body	*z*	m
Distance from the rear wheel to the center of mass	*b*	mm	Pitch angle of vehicle body	*θ*	deg
Half of the front wheel distance	*l_f_*	mm	Lateral angle of the vehicle body	*φ*	deg
Half of the rear wheel distance	*l*_r_	mm	Vehicle mass	*m*	kg

**Table 2:** State-input-output variables overview for full-car.

State	Input	Output
*x*_1_ = z	*u*_1_ = $i_{A}$	*y*_1_ = $\ddot{z}$
*x*_2_ = $\dot{z}$	*u*_2_ = $i_{B}$	*y*_2_ = θ
*x*_3_ = *θ*	*u*_3_ = $i_{C}$	*y*_3_ = φ
*x*_4_ = $\dot{\theta }$	*u*_4_ = $i_{D}$	*y*_4_ = $z_{sA}-z_{wA}$
*x*_5_ = φ	*u*_5_ = $z_{gA}$	*y*_5_ = $z_{sB}-z_{wB}$
*x*_6_ = $\dot{\varphi }$	*u*_6_ = $z_{gB}$	*y*_6_ = $z_{sC}-z_{wC}$
*x*_7_ = $z_{wA}$	*u*_7_ = $z_{gC}$	*y*_7_ = $z_{sD}-z_{wD}$
*x*_8_ = ${\dot{z}}_{wA}$	*u*_8_ = $z_{gD}$	*y*_8_ = $z_{wA}-z_{gA}$
*x*_9_ = $z_{wB}$		*y*_9_ = $z_{wB}-z_{gB}$
*x*_10_ = ${\dot{z}}_{wB}$		*y*_10_ = $z_{sC}-z_{wC}$
*x*_11_ = $z_{wC}$		*y*_11_ = $z_{sD}-z_{wD}$
*x*_12_ = ${\dot{z}}_{wC}$		
*x*_13_ = $z_{wD}$		
*x*_14_ = ${\dot{z}}_{wD}$		

**Table 3:** Input variables in CarSim software.

The Name of the Input Variable	Available Variables
The damping force of the left front suspension	IMP_FD_L1
The damping force of the right front suspension	IMP_FD_R1
The damping force of the left rear suspension	IMP_FD_L2
The damping force of the right rear suspension	IMP_FD_R2

**Table 4:** Output variables in CarSim software.

The Name of the Output Variable	Available Variables
Displacement of the center of mass of the vehicle	Z0
Vertical acceleration of the body center of mass	Az_ SM
Lateral tilt angle	Roll
Lateral angular velocity of the body	AVx
Body roll angle acceleration	AAx
Body pitch angle	Pitch
Pitch angle velocity	AVy
Pitch angle acceleration	AAy
Left front wheel vertical jump speed	Vz_Wc_ L1
Right front wheel vertical jump speed	Vz_Wc_ R1
Left rear wheel vertical jump speed	Vz_Wc_ L2
Right rear wheel vertical jump speed	Vz_Wc_ R2
Left front wheel dynamic load	Fz_ L1
Right front wheel dynamic load	Fz_ R1
Left rear wheel dynamic load	Fz_ L2
Right rear wheel dynamic load	Fz_ R2
Left front suspension dynamic deflection	Cmp D_ L1
Right front suspension dynamic deflection	Cmp D-R1
Left rear suspension dynamic deflection	Cmp D_ L2
Right rear suspension dynamic deflection	Cmp D_ R2

**Table 5:** Parameters of the vehicle.

The Name of the Parameters	Value/Unit
Mass of vehicle	1510 (kg)
Length of vehicle	4600 (mm)
Width of vehicle	1855 (mm)
Height of vehicle	1720 (mm)
The wheelbase of the vehicle	2600 (mm)
Height of the vehicle’s center of mass	650 (mm)
The rotational inertia of the vehicle’s lateral pitch	700.7 (kg/m^2^)
The rotational inertia of vehicle pitching	2059.2 (kg/m^2^)
The inertia of the vehicle’s transverse sway	2059.2 (kg/m^2^)
Distance between the vehicle center of mass and the front axle	1015 (mm)

**Table 6:** Time domain response of vehicle body passing over a triangular speed bump.

Evaluation Indicators		Real Vehicle	CarSim Mode	Deviation
Vertical acceleration of the vehicle body (m/s^2^)	Maximum value	5.2039	5.9550	12.61%
	Minimum value	−5.3627	−5.7855	7.31%
Lateral acceleration of the vehicle body (rad/s^2^)	Maximum value	8.7311	10.3263	15.45%
	Minimum value	−4.8538	−4.5052	7.74%

**Table 7:** Time domain response of the vehicle body passing over a trapezoidal speed bump body.

Evaluation Indicators		Real Vehicle	CarSim Mode	Deviation
Vertical acceleration of the vehicle body (m/s^2^)	Maximum value	8.9954	7.8881	14.04%
	Minimum value	−6.9954	−5.8904	18.75%
Lateral acceleration of the vehicle body (rad/s^2^)	Maximum value	0.5271	0.5457	3.41%
	Minimum value	−1.2644	−1.3568	6.81%

**Table 8:** Comparison of maximum, minimum, and RMS values for the simulation of high-speed driving conditions on Class C roads.

Evaluation Indicators		Passive	LQR	Improvement Volume
Vertical acceleration of the vehicle body (m/s^2^)	Maximum value	0.2010	0.0876	57.89%
	Minimum value	−0.3903	−0.1750	55.16%
	RMS value	0.0818	0.03477	57.48%
Pitch angle acceleration of vehicle body (rad/s^2^)	Maximum value	0.9574	0.7157	25.25%
	Minimum value	−1.0658	−0.7991	25.02%
	RMS value	0.4183	0.2978	28.81%
Dynamic deflection of the front suspension (mm)	Maximum value	16.3211	2.1258	86.97%
	Minimum value	−49.7590	−49.3290	0.86%
	RMS value	27.6931	30.2509	−9.24%
Dynamic deflection of the rear suspension (mm)	Maximum value	14.0317	9.2011	34.43%
	Minimum value	−11.6390	−3.7411	67.86%
	RMS value	6.2951	4.1057	34.78%
Dynamic load of front wheel tires (kN)	Maximum value	6.8297	6.1264	10.30%
	Minimum value	1.9096	3.7112	−94.34%
	RMS value	5.2919	5.2439	0.91%
Dynamic load of rear wheel tires (kN)	Maximum value	4.6055	4.3263	6.06%
	Minimum value	2.8419	3.1677	−11.47%
	RMS value	3.8391	3.8459	−0.18%

**Table 9:** Comparison of the maximum, minimum, and RMS values for the simulation of the double shift line condition with low adhesion coefficient.

Evaluation Indicators		Passive	LQR	Improvement Volume
Vertical acceleration of the vehicle body (m/s^2^)	Maximum value	0.0317	0.0116	63.38%
	Minimum value	−0.0478	−0.0117	75.63%
	RMS value	0.0125	0.0052	58.25%
Pitch angle acceleration of vehicle body (rad/s^2^)	Maximum value	0.1557	0.0738	52.61%
	Minimum value	−0.1771	−0.0813	54.11%
	RMS value	0.0748	0.0334	55.41%
Lateral acceleration of the vehicle body (rad/s^2^)	Maximum value	1.6015	0.4022	74.88%
	Minimum value	−0.7952	−0.6106	23.22%
	RMS value	0.1994	0.1368	31.39%
Dynamic deflection of the left front suspension (mm)	Maximum value	10.3064	7.2013	30.13%
	Minimum value	−43.3650	−36.7230	15.32%
	RMS value	16.4322	16.6863	−1.55%
Dynamic deflection of the left rear suspension (mm)	Maximum value	27.5207	19.7111	28.38%
	Minimum value	−22.1360	−17.8210	19.49%
	RMS value	13.3059	9.8986	25.61%
Dynamic deflection of the right front suspension (mm)	Maximum value	12.8154	10.0643	21.47%
	Minimum value	−49.2730	−49.1270	0.3%
	RMS value	17.5552	18.3658	−4.62%
Dynamic deflection of the right rear suspension (mm)	Maximum value	23.4686	17.8170	24.08%
	Minimum value	−26.7580	−19.9330	25.51%
	RMS value	13.5157	9.8488	27.13%
Dynamic load of the left front tire (kN)	Maximum value	7.1734	6.9756	2.75%
	Minimum value	3.7280	4.2162	−13.10%
	RMS value	5.5553	5.5635	−0.15%
Dynamic load of the left rear tire (kN)	Maximum value	5.9736	5.7911	3.05%
	Minimum value	1.9740	1.9191	2.78%
	RMS value	3.9092	3.8945	0.37%
Dynamic load of the right front tire (kN)	Maximum value	7.0374	7.0032	0.49%
	Minimum value	3.0110	3.2910	−9.30%
	RMS value	5.5534	5.5545	−0.02%
Dynamic load of the right rear tire (kN)	Maximum value	5.5739	5.5905	−0.30%
	Minimum value	1.6679	1.7283	−3.63%
	RMS value	3.9059	3.8916	0.37%

**Table 10:** Comparison of maximum, minimum, and RMS values for emergency acceleration simulation.

Evaluation Indicators		Passive	LQR	Improvement Volume
Vertical acceleration of the vehicle body (m/s^2^)	Maximum value	0.3760	0.2240	40.43%
	Minimum value	−0.5710	−4.9000	14.07%
	RMS value	0.2019	0.0837	58.54%
Pitch angle acceleration of vehicle body (rad/s^2^)	Maximum value	13.2135	5.4922	58.43%
	Minimum value	−2.2410	−3.8202	−70.47%
	RMS value	2.5117	1.1522	54.13%
Dynamic deflection of the front suspension (mm)	Maximum value	−0.0193	−0.0201	−3.83%
	Minimum value	−50.9749	−49.7506	2.4%
	RMS value	31.5615	45.6995	−44.80%
Dynamic deflection of the rear suspension (mm)	Maximum value	66.8743	43.0052	35.69%
	Minimum value	0.0830	0.0841	−1.41%
	RMS value	28.7050	23.0098	19.84%
Dynamic load of front wheel tires (kN)	Maximum value	7.5069	7.6268	−1.60%
	Minimum value	0.3843	0.3390	11.79%
	RMS value	5.0350	4.8221	4.23%
Dynamic load of rear wheel tires (kN)	Maximum value	10.5625	5.6579	46.43%
	Minimum value	1.2721	3.5537	179.36%
	RMS value	4.7952	4.6143	3.77%

**Table 11:** Comparison of maximum, minimum, and RMS values for emergency braking simulation.

Evaluation Indicators		Passive	LQR	Improvement Volume
Vertical acceleration of the vehicle body (m/s^2^)	Maximum value	0.3218	0.0910	71.72%
	Minimum value	−0.3316	−0.2146	35.29%
	RMS value	0.1658	0.0391	76.39%
Pitch angle acceleration of vehicle body (rad/s^2^)	Maximum value	7.1207	0.9671	86.42%
	Minimum value	−3.0148	−1.3838	−54.10%
	RMS value	1.0568	0.3758	64.44%
Dynamic deflection of the front suspension (mm)	Maximum value	2.1514	−11.3811	−429.01%
	Minimum value	−50.0085	−49.0295	1.96%
	RMS value	37.0943	39.8679	−7.48%
Dynamic deflection of the rear suspension (mm)	Maximum value	22.6360	10.6270	53.05%
	Minimum value	−34.8000	−22.7580	34.60%
	RMS value	11.5309	7.3898	35.91%
Dynamic load of front wheel tires (kN)	Maximum value	7.8572	6.5048	17.21%
	Minimum value	2.9680	4.4931	−51.39%
	RMS value	6.0571	6.0092	0.79%
Dynamic load of rear wheel tires (kN)	Maximum value	4.5890	4.0543	11.65%
	Minimum value	2.4109	2.4620	−2.12%
	RMS value	3.3635	3.3620	0.04%

## Data Availability

Not applicable.
